# Radiomics for characterization of the glioma immune microenvironment

**DOI:** 10.1038/s41698-023-00413-9

**Published:** 2023-06-19

**Authors:** Nastaran Khalili, Anahita Fathi Kazerooni, Ariana Familiar, Debanjan Haldar, Adam Kraya, Jessica Foster, Mateusz Koptyra, Phillip B. Storm, Adam C. Resnick, Ali Nabavizadeh

**Affiliations:** 1 Center for Data-Driven Discovery in Biomedicine (D3b), Children’s Hospital of Philadelphia, Philadelphia, PA USA; 2 AI2D Center for AI and Data Science for Integrated Diagnostics, University of Pennsylvania, Philadelphia, PA USA; 3 Department of Neurosurgery, Perelman School of Medicine, University of Pennsylvania, Philadelphia, PA USA; 4 Department of Neurosurgery, Children’s Hospital of Philadelphia, Philadelphia, PA USA; 5 Institute of Translational Medicine and Therapeutics, Perelman School of Medicine, University of Pennsylvania, Philadelphia, PA USA; 6 Division of Oncology, Children’s Hospital of Philadelphia, Philadelphia, PA USA; 7 Department of Pediatrics, University of Pennsylvania Perelman School of Medicine, Philadelphia, PA USA; 8 Department of Radiology, Perelman School of Medicine, University of Pennsylvania, Philadelphia, PA USA

**Keywords:** Oncology, Biomarkers

## Abstract

Increasing evidence suggests that besides mutational and molecular alterations, the immune component of the tumor microenvironment also substantially impacts tumor behavior and complicates treatment response, particularly to immunotherapies. Although the standard method for characterizing tumor immune profile is through performing integrated genomic analysis on tissue biopsies, the dynamic change in the immune composition of the tumor microenvironment makes this approach not feasible, especially for brain tumors. Radiomics is a rapidly growing field that uses advanced imaging techniques and computational algorithms to extract numerous quantitative features from medical images. Recent advances in machine learning methods are facilitating biological validation of radiomic signatures and allowing them to “mine” for a variety of significant correlates, including genetic, immunologic, and histologic data. Radiomics has the potential to be used as a non-invasive approach to predict the presence and density of immune cells within the microenvironment, as well as to assess the expression of immune-related genes and pathways. This information can be essential for patient stratification, informing treatment decisions and predicting patients’ response to immunotherapies. This is particularly important for tumors with difficult surgical access such as gliomas. In this review, we provide an overview of the glioma microenvironment, describe novel approaches for clustering patients based on their tumor immune profile, and discuss the latest progress on utilization of radiomics for immune profiling of glioma based on current literature.

## Introduction

Gliomas are the most common primary brain tumors of all ages. Despite efforts to improve outcome in patients with newly diagnosed glioma, effective treatment remains an unmet need, particularly, in higher grade tumors^1^. Nonetheless, increased application of molecular profiling is providing a better understanding of tumor genomic alterations and has the potential to revolutionize the treatment landscape of glioma through individualized therapies^2,3^. While advancement in immunotherapy has led to the consideration of novel immune-targeted agents for patients with unresectable, metastatic, or recurrent glioma^2,4^, these efforts are still in the early phase. Even with the identification of these novel therapies, significant challenges exist, as in gliomas, vast spatial and temporal intra-tumoral heterogeneity prompts treatment failure^5^.

Increasing evidence suggests that besides mutational and molecular alterations, the immune microenvironment of the tumor also substantially impacts tumor behavior, affecting response or leading to resistance to anti-tumor therapies^6^. Conventional treatments such as radiation and chemotherapy, and novel modalities such as targeted tumor fields and focused ultrasound can also induce alterations within the tumor immune microenvironment (TIME), leading to altered tumor response^7,8^. Therefore, detailed understanding of the TIME is necessary to support the precision medicine approach for improved patient stratification and personalized targeted therapy selection as well as for better application of combination therapies.

Although the standard method for characterizing the tumor immune profile is through performing integrated genomic analysis such as mRNA sequencing on surgical tissue biopsies^9^, the dynamic change in the immune composition of the tumor microenvironment (TME) makes this invasive approach a challenge, especially in the brain tumor space^10^. Also, high-throughput methods are not widely available in many small cancer centers, making this approach unfeasible. Therefore, alternative non-invasive modalities are highly needed for comprehensive and longitudinal evaluation of the tumor immune phenotype through the entire disease course. The recent introduction of artificial intelligence in radiology and the development of “radiomics” have enabled quantitative assessment of images, paving the path for conversion of images into mineable data that convey diagnostic and prognostic information^11^. To date, most studies have used radiomics to predict survival of patients based on multiparametric brain magnetic resonance imaging (mpMRI) but recent advances in machine learning methods are facilitating biological validation of radiomic signatures in glioma and allowing them to “mine” for a variety of significant correlates, including genetic, immunologic and histologic data^12,13^.

Multiparametric MRI-based radiomic signatures hold the potential for non-invasive, serial characterization of the tumor immune phenotype, ultimately aiding in personalized treatment decision-making^14^. Most importantly, as brain MRI is a part of standard clinical work-up for patients with glioma, the method is widely available across large and small institutions. While current use of radiomics as an adjuvant method for immune characterization of tumors may be still limited, the advancement of computational algorithms has the potential to transform the field of precision medicine and to implement radiomics as the primary tool for immune profiling of the TIME in the future^15^.

In this review, we initially provide an overview of the glioma TIME and novel approaches for clustering patients based on their tumor immune profile. We then discuss the latest progress towards using radiomics for imaging-based immune profiling of glioma based on current literature and finally discuss current challenges and future directions in the emerging field of radio-immunomics.

## Characterization of the glioma TIME

### Overview of the glioma immune microenvironment

**Fig. 1:**
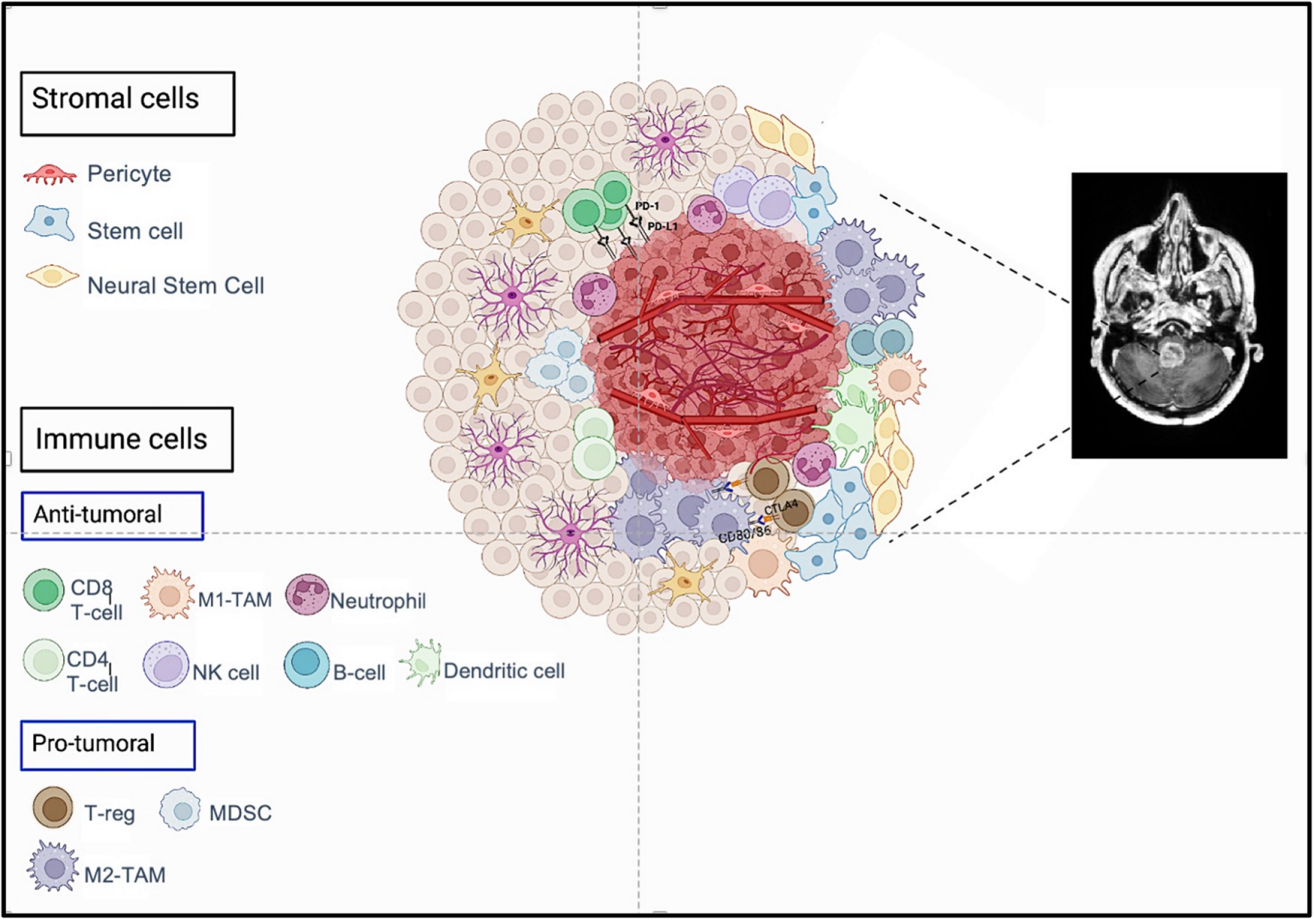
Cellular composition of the glioma microenvironment (created by Biorender.com). The glioma microenvironment is mostly composed of tumor cells, immune cells, and stromal tissue. The most prominent cells of the immune compartment include tumor-associated macrophages, regulatory T-cells, myeloid-derived suppressor cells, T lymphocytes, natural killer cells, and dendritic cells. These cells interact with other components of the tumor microenvironment and play a role in regulating immune response, either through their pro- or anti-tumoral function, subsequently affecting tumor development, progression, and response to therapy. TAMs tumor-associated macrophages, T-reg regulatory T-cells, MDSCs myeloid-derived suppressor cells, NK cell natural killer cell.

In general, the glioma microenvironment is composed of tumor cells, immune cells, and stromal tissue (Fig. 1). The most prominent cells of the immune compartment include tumor-associated macrophages (TAMs), microglia, regulatory T-cells (T-regs), myeloid-derived suppressor cells (MDSCs), T lymphocytes, natural killer cells (NK), and dendritic cells (DCs)^16^. These cells interact with other components of the TME and play a role in regulating immune response to the tumor, either through their pro- or anti-tumoral function, subsequently affecting tumor development, progression, and response to therapy (Fig. 1)^16^. TAMs constitute the largest component of the TIME^16^. Multiple subtypes of TAMs exist, with the most common being M1- and M2-TAMs. Based on the subtype, different interactions are triggered between TAMs and tumor cells; M2-TAMs induce a pro-tumoral microenvironment and promote tumor growth while M1-TAMS induce anti-tumoral effects^17^. Lately, CSF-1R inhibitors such as BLZ945 and PLX3397 have been used to target M2-TAMs^18^. Like TAMs, T-cells compose a major portion of the TIME and have diverse roles. Activated CD8 T-cells, also known as cytotoxic T-lymphocytes, play a major role in anti-tumor immunity. The recruitment, proliferation, and effector function of CD8 T-cell is enhanced by tumor-specific CD4 T-cells that reside within the TIME^19^. Boosting the activity of these T-cell subtypes (CD4 and CD8) has been the target for many immunotherapeutic approaches such as immune checkpoint inhibitors and CAR T-cells^4^. On the other hand, a prominent contributor to the immunosuppressive microenvironment of gliomas is a T-cell subset population commonly named as T-reg. Blocking T-regs through neutralizing antibodies is also being investigated as an approach to inhibit the pro-tumoral effect of T-regs^20^. DCs are potent anti-tumoral cells that generate an immune response through presenting tumor antigens to T cells; due to this ability, DC vaccines, such as DCVax-L, are a major focus of attention for immunotherapy of gliomas^21^. B cells are also efficient antigen-presenting cells that act to enhance clonal expansion of tumor-specific T lymphocytes, boosting anti-tumor response^22^. Other anti-tumoral cells include neutrophils, which are members of the innate immune system. Their presence within the TIME has been associated with the development of resistance to anti-VEGF therapies such as bevacizumab and higher rate of metastasis^23^. NK cells also have an important role in the immune response against tumors and are considered a bridge between the innate and adaptive immune system. In addition to their independent cytolytic activity, they can boost the anti-tumor response induced by CD8 T-cells and DCs in the glioma microenvironment^24–26^. Based on this evidence, numerous NK cell-based immunotherapy trials are being performed for the treatment of glioma^26^. In addition to the cellular compartment, various cytokines (i.e., chemokines, immunosuppressive factors, angiogenic factors) also have an important role in modulating the TIME composition and might have implications for immunotherapy^27^.

Therefore, identifying the most representative genes involved in tumor immune regulation can aid physicians to classify tumors into specific subtypes and thus, yield relevant information regarding the TIME, predicted survival, and immunotherapy responsiveness.

### Approaches for characterization of the TIME

In 2011, a study in patients with colorectal cancer showed that assessment of intra-tumoral immune infiltrates had better performance in predicting tumor recurrence than the conventional TNM staging system^28^. This study set the basis for the concept of stratifying patients based on the characteristics of the TIME such as the density, cell type, functional orientation, and even spatial distribution of immune cells within the tumor.

In the TME, immune and stromal cells are two key tumor-associated normal cells that have been shown to convey diagnostic and prognostic information. Stromal cells have displayed important roles in tumor growth and invasion, as well as to therapeutic resistance^29^. Infiltrating immune cells, however, display a complex interplay and act differently in the context of the tumor type; for example, while tumor-infiltrating lymphocytes (TILS) have demonstrated anti-tumoral effects in ovarian cancer^30^, pro-tumoral properties such as tumor progression and metastasis have been observed with infiltrative immune cells in lung cancer^31^. Besides their prognostic role, tumor-associated normal cells contribute to sample heterogeneity and can impact the molecular analysis of tumor samples by genomic approaches^32^. Thus, computing the infiltration of these non-tumoral cells within the TME, particularly immune cells, provides valuable insight into tumor biology and aids in the development of robust models for determining prognosis and response to therapy.

Over the past decade, several approaches were developed for classification of the tumor immune microenvironment. For example, the ABSOLUTE algorithm estimated the percentage of tumoral cells within the TIME based on somatic DNA copy number alterations, yielding accurate prediction of tumor purity^33^. However, such methods could only predict the tumor composition of samples and did not provide information about the non-tumoral component. The “Estimation of STromal and Immune cells in MAlignant Tumors using Expression data (ESTIMATE)” algorithm aimed to extrapolate tumor cellularity, as well as the proportion of stromal and immune cells present in the TIME of solid cancers by using gene expression markers^34^. For this method, through performing single-sample gene set-enrichment analysis (ssGSEA), four scores were calculated for each tumor sample including the immune score, stromal score, ESTIMATE score and tumor purity score. The immune score was calculated by detecting genes associated with the quantity of infiltrating immune cells in the tumor tissue using leukocyte methylation scores. The ESTIMATE score, defined as the combination of immune and stromal scores, was conversely related with tumor purity. The ESTIMATE algorithm had limited applicability in hematopoietic or stromal tumors (i.e., leukemia and sarcoma) or in tumors with increased fractions of non-tumoral epithelial cells (such as pancreas or prostate cancer) but it showed acceptable performance for immune profiling of many solid tumors, including brain tumors^35,36^.

Later, “Immunoscore” was introduced as a standardized scoring system for the quantification of lymphocyte population; “Immunoscore” evaluated CD3 and CD8 T-cell infiltration both at the tumor core and the invasive margin, subsequently generating a score based on gene expression data^24^. Multiple studies used this method for classifying patients with solid tumors into three groups of “immune hot”, “immune cold” and “immune altered” tumors. In 2018, an international consensus study on patients with colorectal cancer validated the value of the “Immunoscore” among patients with colon cancer, showing that “Immunoscore” has incremental value compared with pathologic T stage, N stage, lymphovascular invasion, tumor differentiation, and microsatellite instability (MSI) status for predicting disease-free survival and recurrence. Furthermore, this immune-based tumor classification demonstrated great value for predicting response to immunotherapy^37^. Nonetheless, as it became apparent that additional cell types other than CD8 and CD3 lymphocytes influence the tumor immune microenvironment, CIBERSORT was introduced as a powerful algorithm that uses gene expression profiles to estimate the proportion of 22 different immune cell types within the TIME^38^.

xCell is another novel gene-signature-based algorithm that benefits from combining gene set enrichment with deconvolution approaches (such as CIBERSORT). This approach consists of identifying gene signatures for 64 cell types, including both immune and stromal cells.

Another novel approach is Tumor Inflammation Signature (TIS), which is a gene signature, composed of 18 genes that measures the level of tumor microenvironment inflammation. This signature has been validated as a gene expression assay that can detect an activated but suppressed adaptive immune response within tumors^39^. TIS was initially introduced in the context of clinical trials as a biomarker that could predict response to immune checkpoint blockades, particularly, pembrolizumab and nivolumab^40^. The ability of TIS in predicting the clinical advantage of anti-PD-1 agents has been shown in different cancer types including melanoma, head and neck squamous cell carcinomas, and triple-negative breast cancers^40^.

Although transcriptome-based cell-type quantification is a more standard and comprehensive method for immune clustering, this approach might not be practical in many minor cancer centers.

Other more feasible approach for assessing the immune composition of TIME is through performing flow cytometry and immunohistochemistry (IHC) analysis. While IHC is more specific and also warrants the preservation of architecture, flow cytometry is more sensitive and is capable of simultaneously assessing numerous markers; hence, these techniques are commonly used as complimentary tools for immunophenotyping^41^. Nevertheless, these techniques also have limitations; for example, flow cytometry requires tissue dissociation process that includes mechanical and enzymatic intervention, which impacts cell viability and therefore introduces potential bias to the readout. Also, the efficacy of routine flow cytometry and IHC assays is hindered by field selection and imprecise semi-quantitative evaluation, novel tools such as digital pathology and image analysis software have been recently established to provide the opportunity for systematic evaluation of the type and density of immune infiltrates in whole-tissue sections^42^.

Table 1 provides a summary and brief comparison of the approaches discussed above.

**Table 1:** Summary of approaches for classification of the tumor immune microenvironment (TIME).

Study	Algorithm	Data type used	Brief description	Advantages	Drawbacks
^33^	ABSOLUTE	DNA copy number array	Estimates percent of tumor cells within the TIME.	Predicts tumor purity.	Only provides information on tumoral components. No information on non-tumoral regions.
^34^	ESTIMATE	Gene expression data	Estimates tumor cellularity and proportion of stromal and immune cells present in TIME.	Provides Immune, Stromal, ESTIMATE, and tumor purity scores. Optimized for solid tumors including brain tumors.	Limited application in hematopoietic and stromal tumors. No quantification of individual immune cell-types.
^28^	Immunoscore	Gene expression data	Quantifies CD3 and CD8 T-cell infiltration at the tumor core and invasive margin.	Used extensively to classify solid tumors as “immune hot, cold, or altered” Validated to have additive value to TNM staging in colorectal cancer. Found to have value in predicting response to immunotherapy.	Limited by cell types assessed. More holistic markers are favored.
^38^	CIBERSORT	Gene expression data	Detects 22 different human immune cell types and provides quantification with high sensitivity and specificity.	Provides a more comprehensive analysis of the TIME. Allow for clustering of immune cell types and correlations of cell types with other biomarkers and signatures.	Not a summary metric.
^96^	xCell	Gene expression data	Identifies 64 different cell types, both immune and stromal using a combination of gene set enrichment and deconvolution approaches.	Provides a large feature set for clustering and correlation. Comprehensive profiling of cell types in the TIME.	Not a summary metric.
^39^	Tumor Inflammation Signature	Gene expression data	Measures level of tumor microenvironment inflammation through the analysis of 18 genes. Originally introduced in the context of clinical trials as a biomarker for immune-checkpoint blockade response.	Provides a metric for inflammatory state. Clinically relevant for novel therapeutics.	Does not provide information on specific immune composition of TIME.

### Identification of specific cell populations of interest

In addition to general characterization of the TIME, a distinct subset of cells that have high clinical significance for identification within the TIME is exhausted CD8+ T-cells. Although tumor-specific CD8 T lymphocytes play a major role in anti-tumor immunity, they are prone to “exhaustion” due to persistent antigenic stimulation. T-cell exhaustion leads to attenuated effector function and immune invasion, resulting in tumor progression in advanced stages^43^. This dysfunctional state is characterized by progressive accumulation of co-inhibitory checkpoints such as CTLA-4, PD-1, and PD-L1. Interestingly, GBM poses a particularly severe T-cell exhaustion signature among infiltrating T-cells compared to other tumor types^44^. Several studies have already applied ssGSEA to identify a gene expression signature for exhausted CD8+ T-cells within the glioma TIME^45,46^. This information is particularly important for overcoming the challenges of immunosuppressive TIME in adult GBM and can help improve the design of novel immunotherapeutic agents and stratify patients for treatments based on this signature.

### Immune classification of the glioma TIME

Several studies have applied CIBERSORT, either alone or in combination with the ESTIMATE algorithm, for classification of immune cell infiltration within the glioma TIME, depicting its ability in predicting patient survival and forecasting response to immune checkpoint inhibitors. For instance, in one study, after identifying the four top immune cells with the highest prognostic value in patients with glioblastoma, four categories of TME scores were developed^47^. Samples with higher TME scores demonstrated a distinct pattern of higher immunological activation genes (i.e., CXCL10, CXCL9) and immune checkpoint expression genes (i.e., PDCD1LG2). Also, survival correlation of the same cohort revealed lower neutrophil and higher CD8 T-cell infiltration to be associated with better prognosis^47^. In another study in patients with glioma, the “immune-high” phenotype, characterized by higher infiltration of the majority of the 22 immune cells, demonstrated higher checkpoint expression but unfavorable prognosis compared with the “immune low” and “immune middle” groups; higher abundance of M0 macrophages, monocytes, M2 macrophages, dendritic cells (activated and resting) and helper T-cells was negatively associated with survival^45^. A similar approach was used for categorizing pediatric glioma into distinct subsets based on immune-related transcriptome profiles^48^. By performing single-sample gene set enrichment analysis (ssGSEA) on 31 immune metagene sets, immunoscores were generated to estimate the overall intra-tumoral immune activity. Unsupervised consensus clustering of the immunoscores identified three distinct clusters: “immune-hot”, “immune-cold”, and “immune-altered” tumors. Through applying the ESTIMATE algorithm, “immune hot” samples demonstrated higher immune and stromal scores while “immune cold” tumors showed lower proportion of immune and stromal cells in the TIME. As expected, the tumor purity score increased from hot to cold tumors while the ESTIMATE score decreased. Correlation of immunoscores with CIBERSORT analysis showed abundance of the majority of the 22 tumor-infiltrating immune cells in “immune hot” tumors with M2-type macrophages, CD4 memory resting T-cells, and resting mast cells being the most identified cells in the TIME. Survival correlation showed that each subset was associated with distinct clinical outcomes, with the “immune hot” tumors showing the best overall survival and the “immune cold” tumors showing the worst prognosis. In addition, an ascending trend of immune checkpoint molecule expression was observed from “immune cold” to “immune hot” tumors, suggesting a possible ability to affect responsiveness to immunotherapies such as anti-PD1 and anti-CTLA4 therapy. These outstanding observations enabled the authors to propose candidate drugs and potential targeted mechanisms for each immune subtype. Another interesting finding was that immune subtypes were correlated with WHO tumor grade, with most of high-grade gliomas (HGG) and diffuse intrinsic pontine glioma (DIPG) being classified as “immune cold”^48^. This finding was also described in another study, which discovered the immune architecture to be subtype-dependent and grade-dependent in pediatric gliomas^49^.

To date, several studies have also utilized IHC and flow cytometry for characterization of the immune infiltrate in glioma^50,51^. In one study, IHC was applied to compare the immune composition between pediatric high-grade and low-grade tumors^52^. In a subset of pediatric glioma samples, IHC analysis demonstrated a trend of higher CD45+, CD8+, and PD1+ cells in low-grade gliomas relative to high-grade tumors, showing a more immunosuppressive TME in low-grade tumors. Through flow cytometry and IHC immunophenotyping, it was concluded that pediatric glioma immune infiltrate is substantially different compared with adult tumors, as in adults, there is evidence of a more robust but immunosuppressive immune infiltrate in high-grade tumors compared to low-grade tumors. Although the validity of these results was limited due to the small sample size, this finding was consistent with the result of larger studies using gene-sequencing methods^53^.

In addition to the above approaches, several studies have also used xCell for immune characterization of the glioma TIME, showing promising results with clinical utility^54–57^.

Overall, identifying the presence and density of various immune cells within the glioma TIME, as well as the most representative genes involved in tumor immune regulation can aid physicians to classify tumors into specific subtypes and yield relevant information regarding predicted survival and therapy responsiveness.

## Radiomics and its role in understanding tumor immune biology

Neuroimaging plays a central role in the diagnosis, treatment planning, and monitoring of brain tumors. With advances in imaging techniques, the amount, variety, and complexity of neuroimaging data acquired during routine work-up of patients with brain tumors has substantially increased. Although traditional radiology practice mostly involves visual interpretation of medical images, the introduction of high-throughput computational methods has changed the paradigm, enabling rapid extraction of innumerable quantitative features from different imaging modalities in a non-invasive and cost-effective manner^11,58^.

**Fig. 2:**
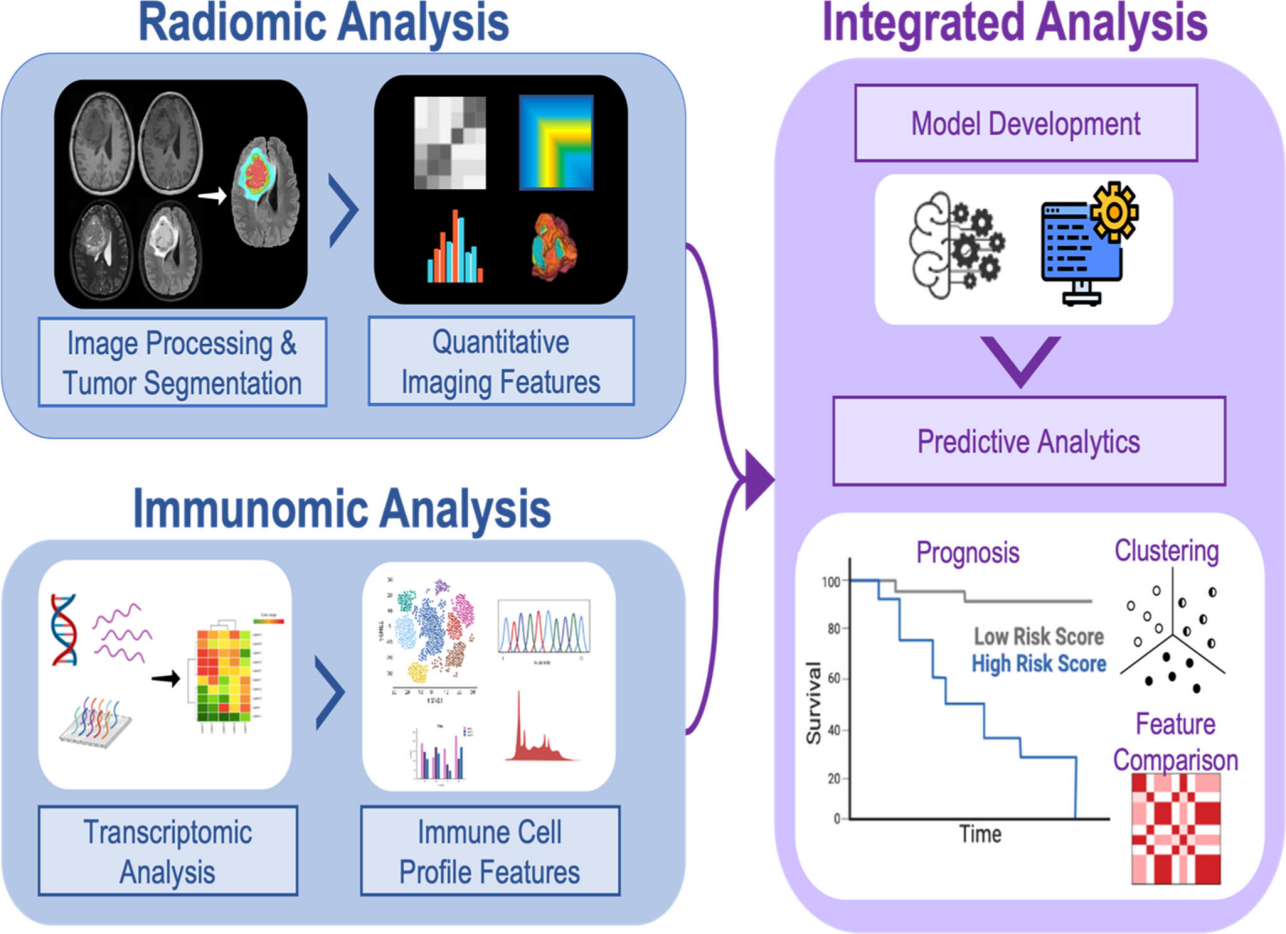
The radio-immunomics workflow (created by Biorender.com). Radiomic analysis: Image pre-processing, tumor segmentation, and radiomic feature extraction is performed in patients with available multiparametric MRI scans. Data analysis is performed for feature selection and radiomics signature construction. Immunomic Analysis: Tumor immune characterization is performed using transcriptomic data. Subsequently, consensus clustering is used to classify patients into distinct subgroups based on their immune profile. Radiomic features and immune data as well as other clinical data are correlated, leading to the discovery of diagnostic and prognostic imaging biomarkers that serve as a substitute for genomic analysis.

Radiomics has shown potential to serve as a non-invasive diagnostic and prognostic tool to capture clinically-relevant, quantitative biologic data from standard and widely-available MRI methods and to discover non-invasive surrogate markers of molecular alterations in glioma samples^59^. With the outstanding pace of precision medicine and patient-tailored targeted therapies, many efforts have shifted towards investigating immune associations with imaging data obtained from different modalities, particularly, MRI scans (Fig. 2)^14^. In the era of immunotherapy, identifying non-invasive biomarkers is an unmet need, especially for cancers with difficult surgical accession such as gliomas. Here, we summarize studies that have been performed to date, demonstrating the utility of radiomics for unraveling various immune-related data in patients with glioma. Since a major area of immunotherapy has been focused on immune checkpoint inhibitors (ICI), most studies have assessed the performance of radiomics in patients receiving ICIs; however, with the emergence of a myriad of other immune-based therapeutics such as vaccines and CAR T-cells^60–62^, radio-immunomics can also have a major impact on non-invasive prediction and surveillance of response to these therapeutic models in addition to ICIs. In the following sections, we summarize the existing studies on the application of radiomics for the prediction of (1) immune cell infiltration, (2) immune signatures and immune-related pathways, and (3) the expression of immune checkpoint inhibitor molecules in patients with glioma. Additional details about these studies can be found in Table 2.

**Table 2:** Studies investigating the association between imaging and immune-related data.

Study	Year	Study size^a^	Tumor grade	Imaging modality	ROI definition	Extracted features	Immune sampling	Database	Findings	Association with survival
*Assessment of immune signatures and immune-related pathways*										
^97^	2014	23	Grade IV	T1, T1CE, T2	Necrosis, edema, infiltrating tumor, enhancing tumor	Morphology + volumetric features	mRNA + DNA microarray analysis	Single-center retrospective study	Absence of mass effect was associated with interleukin 3 and transforming growth factor ß pathways.	n/a
^84^	2014	55	Grade IV	T1, T1CE, T2-FLAIR	Enhancing tumor, necrosis, edema (including non-enhancing tumor)	Morphology + high-order features	Gene expression modules based on various cancer driver genes	TCGA + TCIA (from 4 centers in the US)	Edge blurriness (vs sharpness) of necrotic ROI was positively correlated with IL4 pathway involved in T-cell differentiation and proliferation.	Only enhancement ROI features were associated with survival. (Edge sharpness positively correlated with PFS and OS. Border regularity positively correlated with OS.)
^85^	2016	91	Grade IV	T1, T1CE, T2-FLAIR	Enhancing tumor(CE), necrosis (NE), edema (ED) (including non-enhancing tumor); Tumor bulk (TB) = necrosis + enhancing tumor, Total tumor volume (TV) = edema + tumor bulk	Volumetric features	GSEA	TCGA + TCIA (from 2 centers in the US)	NE, TB, NE/CE, TB/TV were negatively associated with immune response pathways. ED/TV, CE/TB was positively associated with regulation of T-cell activation and proliferation.	NE, CE, TB significantly associated with survival. (CE was the strongest)
^98^	2016	50	Grade IV	T1, T1CE, DWI	Necrotic core, enhancing active tumor, peritumoral edema	Volumetric features + ADC histogram (average mean, standard deviation, skewness, kurtosis and entropy)	n/a	TCGA + TCIA	Mean ADC was significantly negatively correlated with pathways involved in dendritic cell maturation and immune response through following genes: CD4, CD86, MHC class I and class II and MGMT gene.	n/a
^86^	2017	92	Grade IV	T1, T1CE, T2, T2-FLAIR	Edema, enhancing tumor, non-enhancing tumor, necrosis	VASARI features	mRNa and miRNa expression	TCGA + TCIA	T helper cell differentiation, NK cell and B cell activation, interferon gamma response pathways were enriched in less aggressive phenotype (volume-class:T1/FLAIR ratio: hemorrhage ≤2).	Combinatorial phenotype of volume-class, hemorrhage, and T1/FLAIR- ratio significantly stratified survival. A low value for any of these 3 features indicated favorable survival characteristics.
^99^	2018	155 (Training set = 91; Test set = 64)	Grade III	T1, T1CE	Enhancing tumor(CE), non-enhancing tumor	First‐order statistics, shape‐ and size‐based features, textual features	RNA microarray analysis	TCGA + CGGA + TCIA	Genes driving immune system response were significantly enriched in contrast-enhancement regions.	All of the prognostic features were textual features. Seven genes derived from the CE‐specific signature could stratify patients into two subgroups based on overall survival time.
^100^	2018	47	Grade II and III	T2	Whole tumor (abnormal T2 hyperintensity signals)	First-order, textural, wavelet, shape- and size-based features	RNA microarray analysis	CGGA + TCGA + TCIA	High radiomic risk score was associated with immune responses (lymphocyte activation and positive regulation of immune system processes), programmed cell death and, I-kappaB and NF-kappaB signaling.	The radiomic risk score was an independent prognostic factor for PFS and provided significant stratification of PFS in both cohorts.
^101^	2021	95 (Training set = 78; Test set = 17)	Grade IV	T1, T1CE, T2, T2-FLAIR	Solid tumor core, edema; whole tumor (core + edema)	Shape, intensity, texture features	RNA sequencing	TCGA + TCIA + internal data set + external data set	Prognostic radiomics phenotypes were correlated with distinct immune pathway.	Significant association of radiomics signature with overall survival in the training subset (HR = 4.80) and validation subset (HR = 3.68). The C index achieved 0.73 in the training subset and 0.70 in the validation subset.
^69^	2023	149	Grade IV	T1CE, T2	Enhancing tumor, necrosis, edema, peritumoral region (10 mm from the enhanced boundaries of the tumor)	Shape, first-order, textural features, wavelet features	CIBERSORT + ESTIMATE	TCGA + CGGA + CPTAC + TCIA	A model that combined 11 radiomic features was able to distinguish tumors with different Immune cell infiltration (ICI) scores (AUC = 0.96, accuracy = 94%). GBM with a low ICI score exhibits greater necrosis in T1CE and lower expression of the original GLCM texture feature and wavelet feature around the peritumoral regions. GBM with a high ICI score exhibits smaller necrosis in T1CE and higher expression of the original GLCM texture feature and wavelet feature around the peritumoral regions in T2 sequence.	The survival outcomes of patients could be stratified according to the ICI groups predicted by radiogenomic features
*Assessment of specific immune cell subset infiltration*										
^64^	2017	69	Grade IV	T1, T1CE, T2-FLAIR	Enhancing tumor, T2-FLAIR hyperintensity (solid tumor + infiltrating tumor + edema)	Volumetric + textural + intensity features	mRNA expression of CD3D/E/G	TCGA + TCIA	Prediction of CD3 infiltration: Training set: Accuracy = 97.1% and AUC = 0.993. Test set: Accuracy= 76.5% and AUC = 0.847. GLSZM was the best single predictor.	n/a
^66^	2018	60	Grade IV	T2-FLAIR, T1CE, T1, T2, DSC perfusion MRI, DWI	Entire volume of contrast-enhancing lesions, T2 high signal intensity lesions, and necrosis (defined as a hypointense area without contrast enhancement on T1CE within the mass on the FLAIR images)	Volumetrics, mean ADC and CBV values	RNA-level analysis of 14 immune cell markers using quantitative RT-PCR	Single center retrospective study	CD68 (TAMs), CSF1R (TAMs), CD33 (myeloid-derived suppressor cell) and CD4 (helper T-cell, regulatory T-cell) levels were highly positively correlated with nCBV values based on ROIs from both FLAIR and T1CE. CD11b had a significant positive correlation with nCBV values only from T1CE. CD3e (helper T-cell, cytotoxic T-cell) and CD49d showed a significantly negative correlation with ADC. CD33 and CD123, and CD25 were negatively correlated with ADC values from FLAIR and T1CE, respectively. Tumor volumes based on FLAIR or T1CE had significant negative correlations with the expression levels of CD123, CD49d, and CD117, but no immune cell markers showed a significant correlation with tumor necrosis or necrosis ratio.	CD49d was an independent factor for PFS indicating CD49d expression levels correlated with ADC can be a candidate biomarker for predicting progression.
^68^	2020	116 (Training set = 84; Test set = 32)	Grade IV	T1CE, ADC	Total tumor region	First-order statistics, gray-level run length matrix (GLRLM), gray-level co-occurrence matrix (GLCM), shape and size features	RNA sequencing	TCGA, ICGA, TCIA	For T1CE imaging data, the average accuracies of the CTL, aDC, Treg, and MDSC models were 0.72, 0.75, 0.81, and 0.88, respectively. For ADC imaging data, the average accuracies of the aforementioned models were 0.71, 0.61, 0.68, and 0.79, respectively. T1CE features yielded better distinguishability of the enrichment levels of all immune cell subsets relative to ADC features.	The developed radiomics models could reliably identify three immunophenotype groups and aid in the prediction of prognosis.
^102^	2020	107 (Training set = 85; Test set = 22)	Grade II and III	T1, T1CE, T2, T2-FLAIR	Enhancing part of the tumor core, non-enhancing part of the tumor core and peritumoural edema	Intensity, volumetric, histogram-based, and textural features	Tumor Immune Estimation Resource (TIMER) based on RNA sequencing data	TCIA + TCGA	The infiltration levels of B cells, CD8+ T-cells, neutrophils and macrophages estimated by radiomics correlated with those estimated by TIMER in the testing cohort.	n/a
^67^	2021	64	Grade III and IV (GRADE IV, anaplastic oligodendrogliomas, and anaplastic astrocytomas)	T1, T1CE, T2, T2-FLAIR, DSC, ADC	Enhancing tumor and edema (T1CE and FLAIR); whole tumor for rCBV and ADC maps	First-order, shape-based, textural features	Flow cytometry	Single-center retrospective study	The radiomic signatures showed the following diagnostic performance in predicting the immune phenotypes: (1) T-cell fraction (enriched vs deficient), AUC = 0.986; (2) T-cell subclass without Treg (T8 vs T4^*^ dominant), AUC = 0.783; (3) M2-TAM fraction (M2-TAM high vs low), AUC = 0.798. The *IDH*-projected radiomics signature score was significantly positively correlated with the following: M2-TAM; TAM; M2-monocyte/macrophage; and TIL. Regardless of immune phenotype, the majority of the top 10 features were from ADC maps	n/a
^65^	2022	167	Grade II, III, IV	T2	Whole tumor (abnormal hyperintense signals on T2)	First-order, shape and size, textural and wavelet features	Single-cell RNA-sequencing	TCGA + TCIA + internal prospective cohort	The immune system process was significantly related to 14 radiomics features (RFs). Tumor-infiltrating macrophages showed a distinct and strong correlation with prognostic RFs.	Tumor-infiltrating macrophages were highly enriched in patients with higher RF scores and convey poor prognosis.
*Assessment of checkpoint inhibitor expression*										
^72^	2020	85	Grade II, III, IV	T1, T1CE, T2,T2-FLAIR	Enhancing tumor, non-enhancing tumor(NET), peri-tumoral edema (ED)	Intensity, volumetric, morphologic, histogram-based, textural and spatial features	RNA-seq + Gene functional enrichment analysis	TCGA + TCIA	Radiomic features effectively separated gliomas into two subgroups with distinct prognosis: C1 (higher survival) and C2 (lower survival). Patients with C2 radiomic subtype harbored higher CD8 + T-cells, PD1, PD-L1, and CTLA4.	The prognostic value of radiomics extracted from ED region was slightly lower compared with NET and ET region.
^70^	2021	124 (Training set = 68; Test set = 56)	Grade II and III	T1, T1CE, T2, T2-FLAIR	Whole tumor based on FLAIR	n/a	mRNA microarray	TCGA + TCIA + internal cohort	Value of the ROC curve for radiomics-based prediction of IMRiskScore^b^: 0.821 in the test group and 0.708 in the test group.	n/a
*Assessment of prognosis and/or treatment response in immunotherapy trials*										
^74^	2019	22	Grade IV	T1, T1CE, DWI, ADC, DSC perfusion MRI, T2-FLAIR, T2	Tumor volume (enhancing portion of the lesion on T1CE)	Volumetrics, rCBV_max,_ ADCmin	Flow cytometery, ELISA	Single center prospective study	Significant decrease in rADC_min_ was observed after 4 vaccinations only in patients with a persistent increase of natural killer cells (response effectors during immunotherapy) in peripheral blood. Also, difference in cerebral blood volume (ΔrCBV_max_) distinguished TTP from PsP with a sensitivity of 67% and specificity of 75%.	Basal rADC_min_ > 1 significantly predicted longer progression free and overall survival.
^75^	2022	162	Grade IV	T1, T1CE, T2, T2-FLAIR	Enhancing tumor volume	Tumor shape, intensity histogram, and textural features	n/a	Multi-national phase II clinical trial of durvalumab	n/a	Pretreatment radiomics model showed poor performance in predicting OS and PFS. Conversely, first post-treatment radiomics model showed a high C-index for the prediction of OS.

*ROI* region of interest, *GBM* glioblastoma multiforme, *DSC* Dynamic susceptibility contrast, *ADC* Apparent Diffusion Coefficient, *T1CE* T1 post-contrast, *DWI* diffusion-weighted imaging, *TCGA* The Cancer Genome Atlas, *TCIA* The Cancer Imaging Archive, *CGGA* Chinese Glioma Genome Atlas, *ICGA* Indian Cancer Genome Atlas, *CPTAC* The National Cancer Institute’s Clinical Proteomic Tumor Analysis Consortium, *GSEA* Gene set enrichment analysis, *OS* overall survival, *PFS* progression free survival, *CBV* cerebral blood volume, *n*/*a* not available.

^a^The number of patients included in the radiomic analysis.

^b^IMriskScore-related mRNAs are derived from Immunophenotype-associated mRNA signatures and are associated with immune checkpoint expression and prognosis.

### Radiomics for predicting immune cell infiltration in patients with glioma

There are two major lineages of immune cells in TIME that display distinct pro-tumoral or anti-tumoral roles in the tumorigenesis process; the myeloid lineage that includes macrophages, neutrophils, myeloid-derived suppressor cells (MDSC), dendritic cells (DC) and mast-cells, and the lymphoid lineage, which encompasses CD4 helper T-cells, regulatory T-cells (T-regs) and CD8+ cytotoxic T-cells^63^. Thus, a variety of immune cell markers could be used to characterize the immune infiltration of the TIME, which can ultimately help in assessing anti-tumor immune response and informing immunotherapeutic options in clinical trials^7^.

Studies have reported on the association of tumor immunological status in terms of T-cell infiltration with radiomic features and have accordingly generated prognostic radiomic models. In one study, a radiomics model based on six textural features achieved an AUC of 0.847 for predicting CD3 T-cell infiltration among patients with GBM. The authors concluded that image-derived textural diversity might possibly reflect increased immune cell infiltration which increases tumor heterogeneity^64^ (also see Table 2). Since robust T-cell immune response can impact the success of a variety of immunotherapies for glioma patients, assessing CD3 T-cell infiltration through radiomics could provide important data without the inherent risk and sampling limitations of surgery or biopsy.

Tumor-associated macrophages (TAMs) are another major component of the TIME. A recent study aimed to assess the performance of their proposed radiomics model in evaluating the extent of macrophage infiltration and predicting prognosis of patients with glioma. The authors validated their proposed model in a prospective cohort, revealing high enrichment of tumor-infiltrating macrophages and worse prognosis in patients with higher RF scores^65^ (Table 2).

The ability of radiomics in predicting TAM infiltration has also been evaluated using other MRI sequences, such as cerebral blood volume (CBV) mapping with dynamic susceptibility contrast (DSC) perfusion technique. Normalized relative CBV values demonstrated positive correlation with expression levels of TAM markers such as CD68, CSF1R and CD11b in patients with GBM^66^. This finding provides supportive evidence for the assumptive role of TAMs in promoting angiogenesis and tumor invasion.

Radiomic features extracted from ADC maps have also been used to predict the immune cell composition of the TIME. For example, ADC values were reported to be negatively correlated with expression levels of CD49d, CD33, CD123, CD3e, and CD25 (markers of MDSCs, DCs, T-helper cells, CD8 T-cells, and T-regs, respectively)^66^. In another study, radiomic signatures derived from rCBV and ADC maps were able to classify the TIME and potentially assess prognosis based on T-cell fraction (enriched vs deficient), T-cell subclass fraction (CD8 T-cell vs CD4 T-cell dominant), and M2-TAM infiltration fraction (M2-TAM high vs low)^67^ (also see Table 2).

Nonetheless, one study that used T1CE and ADC maps to classify patients with GBM into immune phenotypes showed that although both modalities had acceptable performance, T1CE demonstrated better feasibility in predicting the enrichment levels of all immune cell subsets relative to ADC features^68^.

### Radiomics for the assessment of immune signatures and immune-related pathways

A recent study aimed to establish a prognostic biomarker in patients with GBM^69^, using CIBERSORT to identify various immune cell subsets and the ESTIMATE algorithm to estimate the immune and stromal component, eventually generating an immune cell infiltration (ICI) score. Radiomic analysis was deployed to categorize patients into two groups with high and low ICI scores^69^ (Table 2) and was subsequently validated in an independent cohort. The results of this study suggested that higher ICI scores were indicative of poor prognosis and higher expression levels of multiple immune checkpoint-related genes^69^. This finding can help predict patients who are possibly resistant to single immune checkpoint blockade therapy based on non-invasive radiomics analysis. Another study on patients with diffuse glioma calculated the relative abundance of 16 immune cell infiltrates from RNA-seq data and identified two distinct prognostic radiomic subtypes that were correlated with biological findings. The cluster with more favorable prognosis showed significant enrichment of the genes involved in immune and inflammatory response processes, and upregulation of T-helper cells. On the other hand, the cluster with poorer prognosis was significantly correlated with genes involved in synaptic neurotransmission, and upregulation of T-regs, activated CD8 T-cells, aDCs, neutrophils, and macrophages^70^ (Table 2).

Furthermore, numerous studies have investigated the correlation of radiomic features with immune-related pathways such as immune cell activation and/or maturation. These studies are discussed in Table 2 in detail.

### Radiomics for predicting checkpoint inhibitor expression in glioma

With the growing application of checkpoint inhibitor therapies for treatment of various types of tumors, evaluation of immune checkpoint expression level is essential to predict response to such immunotherapies. As of now, the most widely recognized checkpoint molecules include PD-1, PD-L1, and CTLA-4. Many studies have begun to investigate the utility of radiomics as a non-invasive method for evaluating the expression level of these molecules, which can eventually be used for enrolling patients in clinical trials and predicting response to immune checkpoint inhibitors (Table 2).

“Immunophenoscore” is a biomarker used to predict the tumor immune landscape and to determine response to immunotherapy^71^ and is based on four clusters of immune-related gene sets including checkpoints or immunomodulators. A recent study used deep learning along with radiomic features to develop a model for prediction of risk scores obtained from immunophenoscores in low-grade glioma. Immune checkpoint molecules (including PD-L1, CTLA4, PD1, and LAG3) were significantly enriched in patients with high risk of mortality, i.e., with poor prognosis^70^. Other studies have observed upregulation of PD-1, PD-L1, and CTLA4 mRNA expression levels in radiomic-derived clusters, suggestive of differential responses to immunotherapy in patients categorized in each radiomic subtype^72^.

## Further applications of radiomics in the era of immunotherapy

Besides predicting and classifying patients based on their tumor immune phenotype, radiomics has been extensively investigated for assessing the association between imaging features with immune pathways such as dendritic cell maturation, T-cell activation and proliferation, I-kappaB and NF-kappaB signaling, and interleukin pathways^73^. Table 2 summarizes studies that have reported correlation between radiomic features and immune pathways in patients with glioma.

Also, studies are recently exploring the utility of imaging and radiomics for distinguishing true progression from pseudoprogression in prospective immunotherapy trials^74^. Furthermore, radiomics has been used as a non-invasive method to predict post-treatment survival in patients with glioma receiving immunotherapy^75^ (Table 2).

## Current challenges and future directions

Based on studies performed to date, there is growing evidence that tumor immune profiles can be characterized by radiomic features alone or in combination with other molecular and histological features. Despite great progress, current radiomic findings are generally not mature enough to serve as surrogate predictors of immune biology, as many associations have not yet been thoroughly validated in large sample sizes and external cohorts. Furthermore, most studies have recapitulated immune associations with low complexity and volumetric features, and associations with higher-order statistics remain to be explored. Although there is optimism about the future of this emerging field of radio-immunomics, there are many challenges and limitations to be addressed before it is ready to be used in the clinic. The future of this field is dependent on data sharing and conducting multi-institutional trials.

### Challenges and directions of immune profiling

The whole resected tumor is the ideal specimen for characterization of the tumor immune phenotype. Nevertheless, for the majority of cases, only biopsy samples are available for research purposes. Although being an extremely valuable source for providing information on the tumor, biopsy samples have multiple limitations: being invasive, not being representative of the whole tumor landscape and requiring repetition for evaluating tumor evolution. Liquid biopsy has emerged as a less-invasive approach but due to several factors, including the presence of the blood–brain barrier, its utility is still limited in brain tumors^76^. Also, other less invasive approaches such as anti-CD8 immuno-PET provide limited information regarding the complete immune landscape of the tumor^77^. Lately, there has been interest in en bloc tumor resection or intraoperative MRI to enable spatial navigation and co-registration of MR images and histological slides. However, while possibly beneficial, these studies cannot be performed on all tumors and are associated with technical difficulties^78,79^. Thus, an equally informative alternative to conventional biopsy has yet to be determined for exploring the glioma microenvironment.

Most of the current methods for immune profiling of the biopsy specimen apply bulk gene expression data such as CIBERSORT. Apart from the limitations of biopsy, these techniques are associated with their own set of limitations including sample variability, discrepancy in the RNA extraction step, the impracticality of unequivocally allocating transcripts to specific cell types and differences between the immune phenotypes of distinct cancer types^80^. Unfortunately, novel modalities based on single-cell approaches are costly and unfeasible for large-scale diagnostic use. Nonetheless, MRI-guided biopsy along with genome-scale technology can allow for spatially precise stereotaxic sampling of gliomas to assist RNA-sequencing analysis and yield accurate characterization of intra- and intertumoral clonal heterogeneity. Preliminary studies have been performed in this regard showing promising results^81^.

In addition, a major challenge to immunologic profiling of gliomas is the influence of the complex intra and intermolecular heterogeneity. Previous studies have suggested that molecularly distinct glioma subgroups display distinct microenvironmental landscape, propounding the hypothesis that genetic driver mutations could give rise to unique immunologic profiles. In a large cohort including both pediatric and adult patients with high-grade glioma, immune infiltration patterns were stratified based on mutational and transcriptional profiles to identify subtype-specific immune signatures, independent of age^53^. Interestingly, four distinct patterns of immune cell profiles were identified that correlated with the different transcriptional glioblastoma molecular subgroups. In addition, a meaningful association was observed between immunologic subgroups and overall survival, as well as with immune checkpoint expression^53^. This finding suggests that a multi-omics approach with simultaneous consideration of molecular and immune profiles should be considered in future studies.

Taken together, although the convoluted cellular, molecular, and genetic heterogeneity of brain tumors complicate efforts to define immunologic profiles with clinical applicability, increasing studies are shedding light on the promising path to precision immuno-oncology in glioma.

### Challenges of imaging

Standard MRI sequences, which include pre- and post-contrast T1W, T2W, and T2-FLAIR sequences, help in characterizing the tumor volume and its morphological features. However, many studies have reported findings based on only one or two sequences, limiting the sensitivity and specificity of their results^69^. However, as single biological characteristics may have distinct manifestations on different imaging sequences, there is a need to include additional imaging modalities. Many institutions have taken a step further and are utilizing other advanced MRI techniques such as diffusion-weighted imaging, diffusion tensor imaging, and MR spectroscopy as a useful method for evaluating regions with high cellularity and depicting tumor infiltration in areas of brain that could appear normal on conventional MR images^82,83^. At the current time, the findings from these advanced modalities are limited; thus, time is needed before using imaging data from these modalities as quantitative biomarkers. Furthermore, their widespread application is hindered by variability between imaging acquisition techniques, and post-processing methods.

Another major limitation of imaging is the lack of a consistent definition for specifying certain regions of interest across studies. For example, some studies have defined non-enhancing region as any part of the tumor that does not enhance on T1CE^84,85^ while others have differentiated edema from this “non-enhancing” portion based on abnormal hyperintensity signal on T2W images^86^ (Table 2). Other inconsistencies include whether to incorporate edema into the definition of total tumor volume. Adherence to standard consensus criteria such as RANO and iRANO for adult brain tumors, and RAPNO for pediatric brain tumors can help in applying a unified definition for these regions^87,88^.

### Challenges associated with cohort size and validation

Undoubtedly, one of the most important weaknesses of current studies is the relatively small sample sizes and lack of validation cohorts. Based on our review, ~20% of studies investigating the association of imaging features with immune pathways in glioma had validation cohorts. Also, the mean cohort sizes for primary and validation cohorts were 81 and 38 samples, respectively. It is evident that small patient population without external validation introduces false positive results and limits the generalizability and validity of findings. This indicates a crucial need for multicenter and ideally international collaborations between different institutes via shared resources. Fortunately, through comprehensive and coordinating efforts, consortiums such as The Cancer Genome Atlas (TCGA), The Cancer Imaging Archive (TCIA), Chinese Glioma Genome Atlas (CGGA), and Children’s Brain Tumor Network (CBTN) are attempting to make validation a standard part of ongoing studies^89–91^. Particularly in glioma, due to the vast heterogeneity, access to big data is a crucial need for tackling this challenge.

### Data transparency, reporting, and best practices

The quality of much research in the field of radioimmunnomics is suboptimal due to absent study protocols and inadequate registrations. Also, incomplete reporting and poor data sharing have hindered the transparency of many studies. Setting unified data pooling and protocols, and standard reporting guidelines may promote transparency and good practice.

In this aspect, public genomic databases including TCGA and CGGA, UK Biobank, and open-source repositories for computational analysis tools such as GitHub have been developed to promote transparency and reproducibility of radiogenomic and radioimmunomic studies. An additional effort has been encouraging institutions to register their studies in databases such as Open Science Framework (OSF) to promote data transparency and support practices such as standard study design^92^. It is evident that progress in the field of precision oncology highly relies on initiatives that develop good practice guidelines.

Conclusively, although initial studies have reported promising results, it is vital to develop more standardized and reproducible methods of data interpretation, maintain publicly available databases of radiological studies, and conduct prospective large-scale multi-institutional studies to develop robust models that can be used in clinical trials. In addition, developing agile workflows for image transfer and processing would be crucial for clinical translation of radio-immunomics and will open the door to the exciting opportunity of adding radio-immunomics models to molecular tumor board and provide subject-specific recommendations for treatment based on TIME characterization^93^. Although most studies have been performed in the adult population, the development of this field is particularly important in the context of pediatric gliomas as current standard treatments such as radiation therapy are associated with serious adverse effects such as cognitive decline in pediatric patients. Thus, with increasing preclinical evidence showing the efficacy of immunotherapy for pediatric glioma^94,95^, we recommend further future studies to be focused on this patient population. In conclusion, the emerging field of radio-immunomics can provide an unprecedented opportunity to provide non-invasive tumor-specific characterization of TIME, which can be used to tailor individualized treatment strategies in patients with glioma and to ultimately optimize patient care.

## Data Availability

Data referenced in this review can be accessed by following resources numbered in the “Reference” section.
